# Association between *CFL1 *gene polymorphisms and spina bifida risk in a California population

**DOI:** 10.1186/1471-2350-8-12

**Published:** 2007-03-12

**Authors:** Huiping Zhu, James O Ebot Enaw, Chen Ma, Gary M Shaw, Edward J Lammer, Richard H Finnell

**Affiliations:** 1 Center for Environmental and Genetic Medicine, Institute of Biosciences and Technology, Texas A&M University System Health Science Center, Houston, Texas 77030, USA; 2 Center for Environmental and Rural Health, Texas A&M University, College Station, Texas 77843, USA; 3 California Birth Defects Monitoring Program, Berkeley, CA, USA; 4 Children's Hospital Oakland Research Institute, Oakland, CA, USA

## Abstract

**Background:**

*CFL1 *encodes human non-muscle cofilin (n-cofilin), which is an actin-depolymerizing factor and is essential in cytokinesis, endocytosis, and in the development of all embryonic tissues. *Cfl1 *knockout mice exhibit failure of neural tube closure at E10.5 and die *in utero*. We hypothesized that genetic variation within the human *CFL1 *gene may alter the protein's function and result in defective actin depolymerizing and cellular activity during neural tube closure. Such alterations may be associated with an increased risk for neural tube defects (NTDs).

**Methods:**

Having re-sequenced the human *CFL1 *gene and identified five common single nucleotide polymorphisms (SNPs) in our target population, we investigated whether there existed a possible association between the genetic variations of the *CFL1 *gene and risk of spina bifida. Samples were obtained from a large population-based case-control study in California. Allele association, genotype association and haplotype association were evaluated in two different ethnicity groups, non-Hispanic white and Hispanic white.

**Results:**

Homozygosity for the minor alleles of the SNPs studied (rs652021, rs665306, rs667555, rs4621 and rs11227332) appeared to produce an increased risk for spina bifida. Subjects with the haplotype composed of all minor alleles (CCGGT) appeared to have increased spina bifida risk (OR = 1.6, 95% CI: 0.9~2.9), however, this finding is not statistically significant likely due to limited sample size.

**Conclusion:**

The sequence variation of human *CFL1 *gene is a genetic modifier for spina bifida risk in this California population.

## Background

Neural tube defects (NTDs) are a group of severe congenital malformations characterized by a failure of neural tube closure during early embryonic development. NTDs are complex birth defects with a multi-factorial pattern of inheritance, requiring both genetic and environmental factors to contribute to their etiology [1]. The development and closure of the neural tube is usually completed within 28 days post-conception, in a process that is tightly regulated yet prone to environmental perturbation [2]. Periconceptional folic acid supplementation has been repeatedly reported to prevent 50~70% of NTDs [3-5]. Mutations and polymorphisms in folate pathway genes such as methylenetetrahydrofolate reductase (MTHFR), methionine synthase (MTR), methionine synthase reductase (MTRR), and betaine-homocysteine methyltransferase (BHMT), have been intensively investigated and in some studies have shown an association with NTD risk [6-9]. However, none of these factors individually contributes substantially to the population burden of NTDs.

In addition to folate, other developmental mechanisms have been postulated as contributors to abnormal neural tube development. Animal models have provided crucial mechanistic information and possible candidate genes to explain susceptibility to NTDs [10]. More than 80 genetic mouse models exhibit NTDs, with new ones emerging from gene targeting studies and large-scale mutagenesis screens on a regular basis [2]. A survey of the genes whose disruption causes NTD indicates multiple key signaling pathways and cellular functions that are essential for neural tube closure. One such gene candidate involves non-muscle cofilin (n-cofilin), an actin-depolymerizing factor. N-cofilin, encoded by the *CFL1 *gene, is essential for cytokinesis, endocytosis, and plays a critical role in the development of all embryonic tissues. Inactivation of the *Cfl1 *gene in mice results in embryolethality and failure of neural tube closure by E10.5 [11]. It has been suggested that the neural tube closure defects are due to compromised delamination and migration of neural crest cells in these animals. *In vitro *migration assays performed on neural crest cells from these knockout embryos demonstrated limited traveling distance, failure of cell polarization, and a lack of F-actin structures such as fibers, bundles and cortical F-actins [11]. These findings suggest that n-cofilin regulates cytoskeletal dynamics during neural crest migration.

The human *CFL1 *gene (NM_005507), which maps to chromosome 11q13.1 [12], contains four exons and encodes an 18.5 kDa phosphoprotein. Human n-cofilin protein shares 98.8% homology with mouse protein, and the human *CFL1 *gene is 92.9% homologous with the mouse gene. In this study, we re-sequenced the genomic region on human chromosome 11 which encompasses the *CFL1 *gene, and tested the hypothesis that genetic polymorphisms in human *CFL1 *gene may modify human spina bifida risk. This hypothesis was evaluated in a population-based case-control study of infants with spina bifida.

## Methods

### Subjects

Epidemiological data and biological specimens were derived from the California Birth Defects Monitoring Program, a population-based active surveillance system for collecting information on infants and fetuses with congenital malformations [13]. Program staff collected diagnostic and demographic information from multiple sources of medical records for all live-born and stillborn fetuses (defined as 20 weeks gestation) and pregnancies electively or spontaneously terminated. Nearly all structural anomalies diagnosed within one year of delivery were ascertained. Overall ascertainment has been estimated as being 97% complete [14].

246 cases (infants with spina bifida) and 336 controls (non-malformed infants) were included in this study. Among the 246 cases, 86 (35.0%) were non-Hispanic white, 128 (52.0%) were Hispanic white, and 32 (13.0%) were of other ethnicities (African American, Asian, etc.). Among the 336 controls, 154 (45.8%) were non-Hispanic white, 113 (33.6%) were Hispanic white, and 69 (20.5%) were of other ethnicity (African American, Asian, etc.). These cases and controls were derived from 1983–86 and 1994–95 birth cohorts in selected California counties. Each case and control infant was linked to its newborn bloodspot, which served as the source of DNA in our genotyping analyses. All samples were obtained with approval from the State of California Health and Welfare Agency Committee for the Protection of Human Subjects. Genomic DNA was extracted from dried newborn screening bloodspots using the Puregene DNA Extraction Kit (Gentra, Minneapolis, MN).

### Re-sequencing of CFL1 gene

DNA re-sequencing of *CFL1 *gene was conducted in a subset of samples (48 cases and 48 controls) in order to identify all sequence variations of the target genome region. Primers listed in Table 1 were designed using an online program, Primer3 [15]. The amplicons generated by the primers covered the majority of the *CFL1 *gene locus (GC11M065378: NCBI build 35:11:65378866~65383462), including the complete coding region, 5' and 3' un-translated regions, and all introns. Sequencing analyses were performed using BigDye Terminator Kit version 3.0 (Applied Biosystems, Foster City, CA) on an ABI 3730 Genetic Analyzer (Applied Biosystems, Foster City, CA). Sequencing results were exported to Sequencher™ software version 4.2.2 (Gene Code Corp., Ann Arbor, MI) for alignment and multiple comparisons.

**Table 1:** Primer sequences for re-sequencing of *CFL1 *gene

**Primer set name**	**Primer sequence**	**Size of Amplicon (base pair)**
*CFL*1-1	Fw: 5'-GGAAAAGGGAGAGGAACCAG-3' Rev: 5'-CTGCTACGAGGAGGTCAAGG-3'	538
*CFL*1-2	Fw: 5'-GGGAACTTGGTCTGCTTCAG-3' Rev: 5'-CTGCTTTGATTGGCTCCTTC-3'	455
*CFL*1-3	Fw: 5'-AAGGAGCCAATCAAAGCAGA-3' Rev: 5'-AAGTCTTCAACGCCAGAGGA-3'	666
*CFL*1-4	Fw: 5'-ACATCTGGGTTGACCAGGAG-3' Rev: 5'-AGGGAAGGGGTTGTTGAACT-3'	610
*CFL*1-5	Fw: 5'-ACCAGATGGGACACAACCTC-3' Rev: 5'-GTTGTTTTAGGGCGATTCCA-3'	775
*CFL*1-6	Fw: 5'-GGTCCCTAACCCTGTGGAAT-3' Rev: 5'-TCTAACGAGCTGCGTTCTCA-3'	320
*CFL*1-7	Fw: 5'-TGAGAACGCAGCTCGTTAGA-3' Rev: 5'-GTGCCCTCTCCTTTTCGTTT-3'	695
*CFL*1-8	Fw: 5'-TTCCGGAAACGAAAAGGAG-3' Rev: 5'-TGGGCCTACATTTCCCTACA-3'	271
*CFL*1-9	Fw: 5'-CTGCAGTGCATGTAGGGAAA-3' Rev: 5'-AACTCGAGAGCTGGGTTCAA-3'	611

### Genotyping analysis

Five common single nucleotide polymorphisms (SNPs) were identified from the re-sequenced *CFL1 *gene. SNPs rs652021, rs665306, and rs667555 were genotyped using TaqMan SNP assays (Assay-on-Demand, Applied Biosystems, Foster City, CA) on an ABI PRISM 7900 Sequence Detection System (Applied Biosystems, Foster City, CA) following the manufacturer's instructions. SNPs rs4621 and rs11227332 were genotyped using direct sequencing analyses. Genotyping assays were performed by laboratory technicians who were blinded to the case-control status of the samples. Five percent of the samples were duplicated in order to assess possible genotyping error.

### Statistical analysis

Haploview software version 3.3.2 (Daly Lab at the Broad Institute, Cambridge, MA) was used to perform haplotype analysis [16]. Haploview software predicts haplotype based on EM algorithm. Pair-wise Linkage Disequilibrium (LD) between markers was measured by D' (defined as the linkage disequilibrium measure, D, divided by the theoretical maximum for the observed allele frequencies) and the correlation coefficient r^2^. Spina bifida risk was measured in three ways: allelic association, haplotype association, and genotype association. Single SNP allele association and haplotype association analyses were performed using Haploview software. For each SNP, minor allele frequency (MAF) and heterozygosity (HET) were computed, and deviation from Hardy-Weinberg Equilibrium (HWE) was tested among control infants. For each haplotype, counts for case and control association tests were obtained by summing the fractional likelihoods of each individual. For example, if an individual was determined by the EM algorithm to have a 40% likelihood of haplotype A and 60% likelihood of haplotype B, 0.4 and 0.6 would be added to the counts for A and B respectively. 95% confidence intervals for allele frequencies and haplotype frequencies were calculated using an online program, *VassarStats *[17]. Spina bifida risks were measured by odds ratios (ORs). Haplotype-specific odds ratios were computed taking the most common haplotype as reference. For genotype analysis, ORs were computed for each SNP by logistic regression models utilizing SAS software (version 9.1). Some models were adjusted for race/ethnicity (defined as non-Hispanic white and Hispanic white).

## Results

Five common SNPs were detected through our DNA re-sequencing effort: rs665306 (intron 1), rs667555 (intron 1), rs4621 (exon 2, synonymous, Asp66Asp), rs652021 (intron 2), and rs11227332 (intron 1). The functional effects of these polymorphisms were predicted through FASTSNP (Functional Analysis and Selection Tool for SNP in Large Scale Association Study)[18] and are listed in Table 2. rs4621 is a synonymous SNP on exon 2, and the A allele abolishes a putative exonic splicing enhancer (ESE) domain. rs11227332 is located in intron 1, and the G allele of this SNP disrupts a putative transcription factor (TF) binding site for cAMP-responsive element binding protein (CREB). SNP rs665306 in intron 1 locates in a putative binding site for several transcription factors (TFs), including AP-1, NF-E2 and USF; The nucleotide change from C to T disrupts the putative USF binding site. Another SNP in intron 1, rs667555, is in a putative TF binding site for MZF1, while the less common T allele disrupts the binding. rs652021 is located in intron 2 and has no known functional effect that can be predicted based on current knowledge. One novel G->A change, located 2bp downstream of rs11227332, was observed in 1 spina bifida infant and 1 control infant. Non-synonymous SNPs listed in the NCBI SNP database (Build 36.1), rs11550147, rs11550151, rs11550152, rs11550156, rs11550157, rs11550158 and rs11550160, were not found in our study population. Therefore, these SNPs were not subjected to our analyses of the case-control data.

**Table 2:** Characteristics of *CFL1 *SNP markers and allelic association analysis for spina bifida, non-Hispanic White *vs*. Hispanic White

**dbSNP ID**	**Physical Location (NCBI Build 36.1)**	**Nucleotide Change**	**Location in Gene**	**SNP Function Prediction§**	**Observed Heterozygosity§§**		**HWE p value§§**		**Minor Allele Frequency ** **(95% Confidence Interval)**				**Allelic Association P value**	
					**NHW**	**HW**	**NHW**	**HW**	**NHW**		**HW**		**NHW**	**HW**
									**SB**	**control**	**SB**	**control**		
rs652021	10929142	T/C	intron 2	no known function	0.460	0.438	0.694	0.292	0.412 (0.341,0.487)	0.315 (0.265,0.369)	0.395 (0.337,0.456)	0.446 (0.383,0.512)	0.054	0.293
rs4621	10929314	A/G	exon 2	splicing regulation (protein domain abolished)	0.453	0.432	0.784	0.248	0.406 (0.325,0.481)	0.309 (0.260,0.363)	0.398 (0.339,0.459)	0.446 (0.381,0.512)	0.061	0.334
rs11227332	10929534	A/G	intron 1	intronic enhancer	0.279	0.175	1.000	0.627	0.253 (0.192, 0.326)	0.163 (0.123,0.213)	0.169 (0.128,0.221)	0.107 (0.072,0.156)	0.042	0.057
rs665306	10929820	C/T	intron 1	intronic enhancer	0.459	0.423	0.724	0.184	0.399 (0.328, 0.474)	0.324 (0.274,0.380)	0.394 (0.336,0.455)	0.441 (0.378,0.507)	0.106	0.339
rs667555	10930357	G/T	intron 1	intronic enhancer	0.466	0.435	0.540	0.332	0.386 (0.315, 0.461)	0.321 (0.266,0.372)	0.391 (0.333,0.453)	0.425 (0.361,0.492)	0.161	0.509

**§: **SNP function prediction performed using *FASTSNP *(Functional Analysis and Selection Tool for SNP in Large Scale Association Study); **§§: **Computed in control group

**NHW: **non-Hispanic white; **HW: **Hispanic white; **HWE: **Hardy-Weinberg Equilibrium; **SB: **spina bifida;

The five common SNPs were genotyped in the case-control study. Genotyping was successful for 99.6%, 99.2%, 95.1%, 98.0% and 96.7% among cases, and 99.1%, 98.8%, 86.9%, 97.0% and 94.9% among controls, for rs652021, rs4621, rs11227332, rs665306 and rs667555, respectively. Missing of genotyping results was due to failure of PCR amplification caused by limited amount of DNA. Successful rates between cases and controls were not significantly different except for SNP rs11227332. Characteristics of SNP markers genotyped for *CFL1 *gene as well as allelic association with spina bifida risk in the two major subpopulations, non-Hispanic whites and Hispanic whites, are listed in Table 2 and sorted by their physical location on the chromosome. Among controls, non-Hispanic whites and Hispanic whites were in Hardy-Weinberg Equilibrium (HWE) for all SNPs studied. Strong linkage disequilibria were observed for all markers in both populations based on the calculation of D' and r^2 ^(Table 3). Among the non-Hispanic whites, SNPs rs652021, rs4621 and rs11227332 presented significantly higher minor allele frequencies (MAF) in the spina bifida infants than in the controls (p < 0.05), with no appreciable difference between the Hispanic white cases and controls; this suggests a possible association between these less common alleles and spina bifida risk among the non-Hispanic white population.

**Table 3:** Pairwise LD of *CFL1 *SNP markers in different ethnicities

**nonHispanic White**					
	**rs652021**	**rs4621**	**rs11227332**	**rs665306**	**rs667555**

**rs652021**	--	0.985	0.335	0.954	0.732
**rs4621**	1.000	--	0.342	0.939	0.746
**rs11227332**	0.879	0.88	--	0.381	0.391
**rs665306**	0.984	0.984	0.958	--	0.744
**rs667555**	0.856	0.87	0.959	0.869	--

**Hispanic White**					

	**rs652021**	**rs4621**	**rs11227332**	**rs665306**	**rs667555**

**rs652021**	--	0.982	0.093	0.982	0.89
**rs4621**	1.000	--	0.095	0.964	0.872
**rs11227332**	-1.000	-1.000	--	0.092	0.088
**rs665306**	1.000	1.000	-1.000	--	0.888
**rs667555**	0.980	0.979	-1.000	0.96	--

D' is below the diagonal, r^2 ^is above the diagonal

We performed association analyses between haplotypes and spina bifida risk in the two major ethnicity groups using the Haploview program; the results are presented in Table 4. Haplotypes were estimated based on the EM algorithm. Only haplotypes with frequencies greater than 1% were included in the analyses. In non-Hispanic white controls, the most common haplotype was TAACG (0.642), while in Hispanic white controls, the most common haplotype was CGATT (0.441). In both ethnic groups, the haplotype CGGTT, which is composed of all minor alleles for the five SNPs, appeared to be associated with a slightly increased risk for spina bifida (OR = 1.6). We then tested haplotypes of each two adjacent SNPs. In non-Hispanic whites, haplotype CG for rs652021-rs4621 (OR = 1.5, 95% CI: 1.0, 2.2) and haplotype GT for rs11227332-rs665306 (OR = 1.6, 95% CI: 1.0, 2.6) appeared to be associated with modestly increased risk of spina bifida compared to wild types TA or AC, respectively. In Hispanic whites, haplotype GT for rs11227332-rs665306 exhibited slightly elevated risk (OR = 1.6, 95% CI: 0.9, 2.9).

**Table 4:** Associations between haplotypes and spina bifida risk, non-Hispanic White *vs*. Hispanic White

**Haplotype**	**Freq. (95% CI) §**	**Estimated Haplotype Counts**		**OR**	**95% CI**
		**Case**	**Control**		

**Non-Hispanic White**					
TAACG	0.642 (0.588,0.695)	96.7	192.5	ref.	
CGGTT	0.161 (0.123,0.206)	38.7	48.4	1.6	0.9, 2.6
CGATT	0.128 (0.094,0.169)	24.1	38.3	1.3	0.7, 2.2
CGATG	0.028 (0.009,0.075)	8.3	3.2	--	--
TAACT	0.024 (0.011,0.047)	1.1	7.3	--	--
**Hispanic White**					
CGATT	0.441 (0.376,0.505)	108.7	98.7	ref.	
TAACG	0.414 (0.354,0.483)	96.9	92.8	0.9	0.6, 1.4
CGGTT	0.108 (0.073,0.155)	43.2	24.2	1.6	0.9, 2.9
TAACT	0.023 (0.010,0.051)	3.1	5.2	--	--

**Non-Hispanic White**					
**Block 1**	**rs652021-rs4621**				
TA	0.683 (0.629,0.733)	99.0	209.0	ref.	
CG	0.314 (0.264,0.368)	68.0	96.0	**1.5**	**1.0, 2.2**
**Block 2**	**rs11227332-rs665306**				
AC	0.668 (0.614,0.720)	100.6	201.7	ref.	
AT	0.162 (0.124,0.207)	27.2	48.3	1.1	0.7, 1.9
GT	0.165 (0.129,0.213)	41.0	50.4	**1.6**	**1.0, 2.6**
**Hispanic White**					
**Block 1**	**rs652021-rs4621**				
TA	0.442 (0.379,0.508)	100.0	100.0	ref.	
CG	0.553 (0.488,0.617)	153.0	125.0	1.2	0.9,1.8
**Block 2**	**rs11227332-rs665306**				
AT	0.456 (0.394,0.523)	112.1	102.6	ref.	
AC	0.438 (0.375,0.503)	100.5	99.0	0.9	0.6, 1.4
GT	0.106 (0.074,0.156)	43.4	24.4	1.6	0.9, 2.9

**§ **Estimated haplotype frequencies and 95% confidence intervals in control group

We also evaluated the association between individual *CFL1 *SNP marker genotypes and spina bifida risk (Table 5) in the overall population, as well as in the two major ethnic subpopulations, non-Hispanic white and Hispanic white. Among non-Hispanic whites, homozygotes for the minor alleles for all SNPs exhibited more than a two-fold increase in risk for spina bifida. Increases in risk were also seen in Hispanic whites; however, these increases were not statistically significant, i.e., consistent with random variation.

**Table 5:** Association between *CFL1 *genotypes and spina bifida risk: Odds Ratio estimation by Logistic Regression

	**No. of Control (%)**	**No. of Spina Bifida (%)**	**Crude OR (95% CI)**	**Non-Hispanic White OR (95% CI)**	**Hispanic White OR (95%CI)**
**rs652021**					
TT	102 (30.6)	55 (22.5)	ref	Ref	ref
TC	154 (46.3)	122 (49.8)	1.5 (0.9, 2.2)	1.1 (0.6, 2.0)	1.8 (0.9, 3.7)
CC	77 (23.1)	68 (27.8)	**1.6 (1.0, 2.6)**	**2.8 (1.2, 6.5)**	1.7 (0.8, 3.6)
**rs4621**					
AA	104 (31.3)	57 (23.4)	ref	Ref	ref
AG	151 (45.5)	119 (48.8)	1.4 (0.9, 2.2)	1.1 (0.6, 2.0)	1.7 (0.9, 3.5)
GG	77 (23.2)	68 (27.9)	**1.6 (1.0, 2.6)**	**2.8 (1.2, 6.5)**	1.6 (0.8, 3.3)
**rs11227332**					
AA	224 (76.7)	162 (69.2)	ref	ref	ref
AG	61 (20.9)	58 (24.8)	1.3 (0.9, 2.0)	1.3 (0.7, 2.4)	1.6 (0.8, 3.0)
GG	7 (2.4)	14 (6.0)	**2.8 (1.1, 7.0)**	**5.1 (1.3, 20.2) §**	2.8 (0.6, 14.4)
**rs665306**					
CC	98 (30.1)	57 (23.7)	ref	ref	ref
CT	151 (46.3)	117 (48.6)	1.3 (0.9, 2.0)	1.0 (0.6, 1.9)	1.9 (0.9, 3.8)
TT	77 (23.6)	67 (27.8)	1.5 (0.9, 2.4)	**2.3 (1.0, 5.2)**	1.7 (0.8, 3.5)
**rs667555**					
GG	96 (30.1)	55 (23.1)	Ref	Ref	ref
GT	148 (46.4)	122 (51.3)	1.4 (0.9, 2.2)	1.1 (0.6, 2.0)	1.9 (0.9, 4.0)
TT	75 (23.5)	61 (25.6)	1.4 (0.9, 2.3)	2.2 (0.9, 5.5)	1.6 (0.7, 3.4)

**OR**: Odds Ratio; **95% CI**: Wald 95% Confidence Interval;

**§**: The smallest cell n = 3, Fisher Exact p = 0.01.

We performed functional prediction using an online program, *FASTSNP*, which predicts functional impact of SNPs according to current knowledge. FASTSNP provides a "risk score" for each SNP based on its putative biological function. Among the five SNPs we studied, rs4621, which is a synonymous change located in exon 1, has the highest "risk score". SNP rs652021 has a risk score of zero, which means that it has essentially no known functional impact. Strong linkage disequilibrium (LD) was observed among the five SNPs in both sub-populations (non-Hispanic white and Hispanic white) in our study. Therefore, the observed associations of these SNPs with spina bifida could be due to the strong LD to the "real" functional variation.

## Discussion and conclusion

To our knowledge, this is the first investigation of human *CFL1 *gene polymorphisms as risk factors of spina bifida risk. Infants with the CGGTT haplotype had an increased spina bifida risk in the two studied ethnic groups. The observed increases in spina bifida risks indicated a possible role of actin depolymerizing factor in human neural tube morphogenesis.

One possible mechanism underlying the observed results that can be suggested follows. During early embryonic development, genes that regulate various morphogenetic activities must function cooperatively for the neural tube to close properly. Neural crest cell migration is one of these critical activities [2]. Neural crest cells migrate from the neural tube to the periphery and give rise to a variety of cell types. Therefore faulty neural crest cell migration might interfere with normal neural tube closure. This suggestion is bolstered by observations derived from experimental models. Mammals have at least three highly conserved genes encoding F-actin polymerizing factors: n-cofilin [19], m-cofilin (muscle cofilin) [20], and ADF (actin depolymerizing factor) [21]. Actin depolymerization is one of the key activities required for actin-driven motility [22]. Recently, detailed analysis of n-cofilin function in mouse embryonic development using gene knockout technology suggested that n-cofilin is essential for neural crest cell migration [11]. Our study, from a population perspective, revealed that genetic variations of actin depolymerizing factor n-cofilin among infants may contribute to the risk of spina bifida.

The strengths of this study include its population-based ascertainment of cases and controls and its evaluation of race/ethnicity as a potentially important modifier of risk in the presence of variant genotypes and haplotypes. Population variation needs to be evaluated in population-based case control studies of gene-risk association. Haplotype association analysis in both sub-populations suggested that haplotype CGGTT, which is comprised of minor alleles of all five SNPs, conferred an increased risk of spina bifida. Although not unexpected, it is noteworthy that the distribution of haplotypes differed between the two ethnic groups. For example, the frequency of the high risk haplotype CGGTT in non-Hispanic whites (0.161) was higher than that in Hispanic whites (0.108). Ordinal logistic regression analysis revealed that the homozygous status of minor alleles of all five SNPs were each associated with a greater than two-fold increase in spina bifida risk in the non-Hispanic white population. These increases were also seen in Hispanic whites, even though they did not reach statistical significance. There is currently no experimental data on the functional consequences of these variants. We performed functional prediction using an online program, *FASTSNP*, which predicts functional impact of SNPs according to current knowledge. FASTSNP provides a "risk score" for each SNP based on its putative biological function. Among the five SNPs we studied, rs4621, which is a synonymous change located in exon 1, has the highest "risk score". SNP rs652021 has a risk score of zero, which means that it has essentially no known functional impact. Strong linkage disequilibrium (LD) was observed among the five SNPs in both sub-populations (non-Hispanic white and Hispanic white) in our study. Therefore, the observed associations of these SNPs with spina bifida could be due to the strong LD to the "real" functional variation.

Our results, although based on multiple tests on small sample sizes and therefore lowered statistical power, represent a preliminary step in elucidating the association between *CFL1 *gene variations and spina bifida risk. The haplotype CGGTT, which is composed of minor alleles of all five SNPs and was commonly found in both major race/ethnic groups studied, conferred an increased risk of spina bifida. Combined with existing knowledge about NTDs, these polymorphisms could clarify the complex mechanism of genetic susceptibility underlying the risk for NTDs. Further studies of these variations using cellular experiments could potentially provide an in-depth view of the mechanism of our observations.

## Competing interests

The author(s) declare that they have no competing interests.

## Authors' contributions

HPZ designed the study, optimized and performed part of the experiments and wrote the manuscript; JOE performed the majority of the experiments; CM performed the statistic analyses; GMS performed epidemiology study design and helped with manuscript writing; EJL helped with study design and manuscript writing; RHF performed experimental design and helped with manuscript writing. All authors read and approved the final manuscript.

## Pre-publication history

The pre-publication history for this paper can be accessed here:


